# P-63. Real-World Effectiveness of *Influenza* Vaccine Over a Decade During the 2011–2021 Seasons: Implications of Vaccine Mismatch

**DOI:** 10.1093/ofid/ofae631.270

**Published:** 2025-01-29

**Authors:** Yu Jung Choi, Joon Young Song, Seong-Heon Wie, Won Suk Choi, Jacob Lee, Jin-Soo Lee, Young Keun Kim, Shin-Woo kim, Sun Hee Lee, Kyung-Hwa Park, Hye Won Jeong, Jin Gu Yoon, Hye Seong, Eliel Nham, Ji Yun Noh, Hee Jin Cheong, Woo Joo Kim

**Affiliations:** Division of Infectious Diseases, Department of Internal Medicine, Guro Hospital, Korea University College of Medicine, Seoul, South Korea, Seoul, Seoul-t'ukpyolsi, Republic of Korea; Division of Infectious Diseases, Department of Internal Medicine, Korea University College of Medicine, Seoul, South Korea, Seoul, Seoul-t'ukpyolsi, Republic of Korea; Division of Infectious Diseases, Department of Internal Medicine, St. Vincent Hospital, College of Medicine, The Catholic University of Korea, Seoul, Korea, Kyonggi-do, Kyonggi-do, Republic of Korea; Korea University Ansan Hospital, Ansansi, Kyonggi-do, Republic of Korea; Division of Infectious Diseases, Department of Internal Medicine, Kangnam Sacred Heart Hospital, Hallym University College of Medicine, Seoul, South Korea, Seoul, Seoul-t'ukpyolsi, Republic of Korea; Division of Infectious Diseases, Department of Internal Medicine, Inha University School of Medicine, Incheon, South Korea, Incheon, Inch'on-jikhalsi, Republic of Korea; Yonsei University Wonju College of Medicine, Wonju, Kangwon-do, Republic of Korea; School of Medicine, Kyungpook National University, Daegu, Taegu-jikhalsi, Republic of Korea; Division of Infectious Disease, Department of Internal Medicine, Pusan National University Hospital, Seo-gu, Pusan-jikhalsi, Republic of Korea; Division of Infectious Diseases, Department of Internal Medicine, Chonnam National University Medical School, Gwangju, South Korea, Gwangju, Cholla-namdo, Republic of Korea; Department of Internal Medicine, Chungbuk National University Hospital, Cheongju, Republic of Korea / Department of Internal Medicine, Chungbuk National University College of Medicine, Cheongju, Republic of Korea,, Cheongju, Ch'ungch'ong-bukto, Republic of Korea; Division of Infectious Diseases, Department of Internal Medicine, Korea University College of Medicine, Seoul, South Korea, Seoul, Seoul-t'ukpyolsi, Republic of Korea; Division of Infectious Diseases, Department of Internal Medicine, Korea University College of Medicine, Seoul, South Korea, Seoul, Seoul-t'ukpyolsi, Republic of Korea; Division of Infectious Diseases, Department of Internal Medicine, Korea University College of Medicine, Seoul, South Korea, Seoul, Seoul-t'ukpyolsi, Republic of Korea; Division of Infectious Diseases, Department of Internal Medicine, Guro Hospital, Korea University College of Medicine, Seoul, South Korea, Seoul, Seoul-t'ukpyolsi, Republic of Korea; Division of Infectious Diseases, Department of Internal Medicine, Korea University College of Medicine, Seoul, South Korea, Seoul, Seoul-t'ukpyolsi, Republic of Korea; Division of Infectious Diseases, Department of Internal Medicine, Korea University College of Medicine, Seoul, South Korea, Seoul, Seoul-t'ukpyolsi, Republic of Korea

## Abstract

**Background:**

Influenza imposes a significant healthcare burden in Korea, leading the government to initiate a national immunization program. Previous studies on vaccine effectiveness (VE) were limited to single-season estimation in Korea.

Matching status of epidemic influenza virus strains and vaccine strains in each season from the 2011/2012 to 2020/2021 seasons

**Figure:**
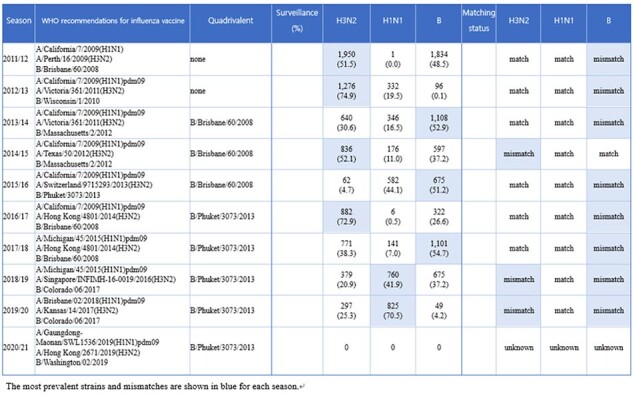

**Methods:**

This multicenter prospective cohort study enrolled patients with influenza-like illnesses at 10 medical centers in Korea from 2011–2021. The demographic and clinical data were collected from questionnaire surveys and electronic medical records. Using a test-negative design, we aimed to investigate the effectiveness of a seasonal influenza vaccine for antigenic matching of the vaccine and circulating viral strains over 10 seasons.

Vaccine effectiveness against laboratory-confirmed influenza

**Figure:**
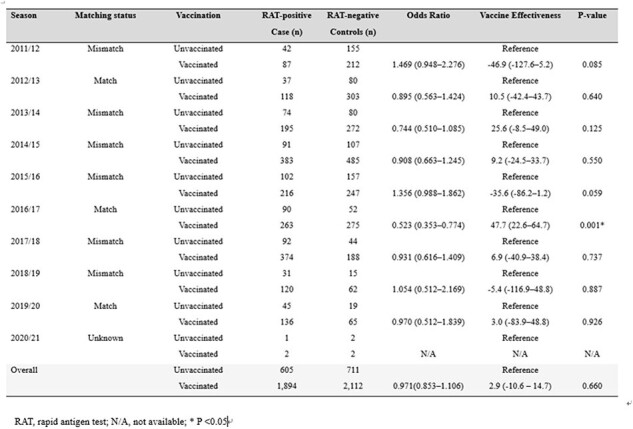

**Results:**

Overall, 5,322 adults aged ≥65 years were enrolled. Only three (33.3%) of nine seasons showed >70% antigenic match between vaccine and circulating strains. Influenza VE was significantly variable by season, ranging from -46.9% (95%confidence interval [CI]: -127.6–5.2) in the 2011/12 season to 47.7% (95%CI: 22.6–64.7) in the 2016/17 season. A significant difference was observed in the VE depending on whether the vaccine strains matched with epidemic strains: 28.8% (95%CI: 8.8–44.8) in matched seasons versus -12.0% (95%CI: -30.0–3.7) in mismatched seasons. Across the study period, influenza-related hospitalizations were reduced by 13.6% (95%CI: 0.7–24.8) with vaccination. In a subgroup analysis, the VE against influenza-related hospitalization was 48.4% (95%CI 29.6–62.2) in A/H3N2 dominant seasons and 53.8% (95%CI: -73.4–87.7) in A/H1N1 dominant seasons, respectively.

Vaccine effectiveness against laboratory-confirmed influenza by the matching status of vaccine and epidemic strains

**Figure:**
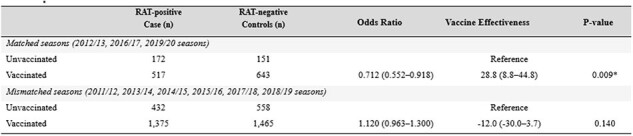

**Conclusion:**

Influenza vaccine mismatch was frequent over the study period, leading to negligibly low VE in mismatched seasons. Influenza vaccination reduces the risk of influenza-related hospitalizations.

Estimates of vaccine effectiveness for influenza-related hospitalizations

**Figure:**
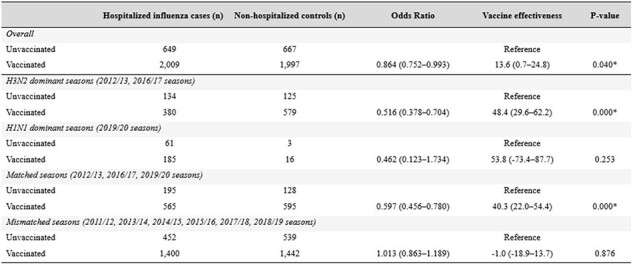

**Disclosures:**

**All Authors**: No reported disclosures

